# Giant local circular dichroism within an asymmetric plasmonic nanoparticle trimer

**DOI:** 10.1038/srep08207

**Published:** 2015-02-03

**Authors:** Hancong Wang, Zhipeng Li, Han Zhang, Peijie Wang, Shuangchun Wen

**Affiliations:** 1 Key Lab of Micro-/Nano- Optoelectronic Devices of Ministry of Education, School of Physics and Electronics, Hunan University, Changsha 410082, China; 2 The Beijing Key Laboratory for Nano-Photonics and Nano-Structure (NPNS), Center for Condensed Matter Physics, Department of Physics, Capital Normal University, Beijing 100048, China; 3 SZU-NUS Collaborative Innovation Center for Optoelectronic Science & Technology, Key Laboratory of Optoelectronic Devices and Systems of Ministry of Education and Guangdong Province, College of Optoelectronic Engineering, Shenzhen University, Shenzhen 518060, China

## Abstract

We investigated the near-field response in silver nanoparticle aggregates to the excitation of circular polarized light. In a right-angle trimer system, the local field intensity excited by right-hand circularly polarized light is almost one thousand times larger than the left-hand case. By analyzing the polarization and phase of the local field in plasmonic hotspots, we found this local circular dichroism is originated from the near-field interference excited by orthogonal polarized incident lights. The local circular dichroism can be tuned by the rotation of the third particle, the interparticle distance, and the dielectric environment. This phenomenon could also widely exist in more complicated nanoaggregates. These findings would benefit for resolving light handedness, and enhancing circular dichroism and optical activity.

Chiroptical effects are typically characterized by small differences of extinction coefficients or refractive indexes in the interaction of left-hand circularly polarized light (LCP) and right-hand circularly polarized light (RCP) with chiral molecules, leading to circular dichroism (CD) or optical activity (OA)^1^. The CD spectroscopy is widely used to investigate the subtle structural information of organic and biological molecules^2^. High concentration and large quantity of chiral specimens are usually demanded for reliable signal-to-noise. Concepts borrowed from molecular science, giant CD or OA response have been observed in metallic nanostructures such as spirals^3^, crosses^4^,^5^,^6^, nanorods^7^, helixs^8^,^9^, oligomers^10^,^11^, pyramidals^12^, *etc*^13^. The mechanism of either exciton-plasmon interaction between chiral molecules and metal nanostructures^14^,^15^, or near-field interactions in plasmonic nanostructures with chiral geometry^16^,^17^,^18^, makes the CD or OA response much stronger than the one from merely chiral molecules. The surface enhanced Raman optical activity (SEROA) of chiral molecule is contributed to the plasmonic hotspots in metal nanoparticles^19^,^20^. The change of symmetry in nanoaggregates would alter the polarization response of Raman scattered light^21^. Also, new chiral center might generate for different sorption sites in aggregates, which makes it challenging to obtain the stability and repeatability of SEROA. Recently, it was found even for an achiral molecule in a planar nanoparticle trimer, Raman scattering of the molecule can carry a considerable degree of circular polarization, that is, scattered circular polarization Raman optical activity (SCP ROA)^22^,^23^. The origin of this plasmonic ROA in the molecule-aggregates system is also the nanoantenna effect of asymmetric particles excited by the Raman emission of the molecule^24^. The single-molecule ROA may open a new perspective on the characterization for structural biology and pharmaceutical industry^25^. Actually, local field in the nanogap of aggregates could also have CD characteristic, which might result in incident circular polarization ROA^26^,^27^ and local scattering OA^28^. For example, the large scattering OA has been locally observed in random fractal aggregates of silver nanoparticles by photon scanning tunneling microscopy. OA properties of aggregates have been simulated to reproduce the experiment observations^28^,^29^,^30^,^31^. However, in the complicated coupled nanoparticles system^32^,^33^,^34^,^35^, a fundamental insight into the relation between the near field and geometry under the excitation of circularly polarized light (CPL), is still needed for deeply understanding the origination of these OA phenomena, which will also be beneficial for the design of plasmonic CD or ROA nanostructures.

In this paper, the local field response to the CPL excitation in nanoparticle dimer and trimer was investigated. We found the near-field enhancement in an asymmetric trimer is highly dependent on the polarization rotation. The local field at a hotspot excited by RCP is much larger than the LCP case. The dependence originates from the near-field interference in the strong coupled nanoantenna system and can be tuned by the rotation of the third particle, the interparticle distance, and the dielectric environment. Similar to far-field CD, we introduce a parameter *ρ* in order to evaluate the local CD. Both positive and negative *ρ* are found in the asymmetric nanoantenna system. Besides, *ρ* is very sensitive to the environment. This study could provide a more flexible way to design plasmonic nanostructures with strong CD or ROA response for various applications such as polarization sensitive devices^3^,^8^ and plasmonic sensors^36^,^37^.

## Results

**Figure 1:**
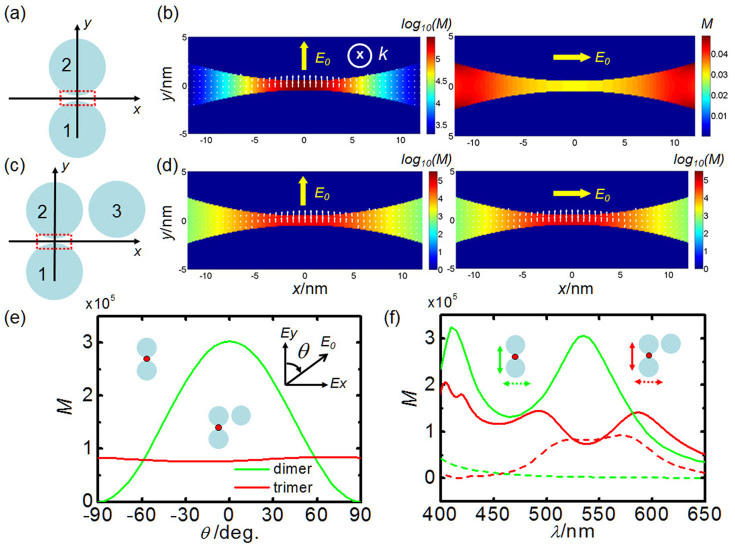
Near-field enhancements in silver nanoparticle aggregates. Schematic diagrams of the dimer (a) and the right-angle trimer (c). The radius of particles are all 40 nm with the interparticle distance *d* = 1 nm. (b) Electric field intensity distributions around the interparticle junction of a dimer under parallel and perpendicular polarized excitations at *λ* = 532 nm. White arrow shows the direction of electric field. (d) Electric field intensity distributions around the 1^st^–2^nd^ interparticle junction in the trimer with the same excitations as Fig. 1b. (e) Local field intensity at the hotspot in the interparticle junction (denoted by red dot in the inset) of the dimer (green curve) and trimer (red curve) as the function of the angle of the incident polarization, respectively. (f) Local field intensity in the dimer (green curves) and trimer (red curves) as a function of wavelength for parallel (solid curves) and perpendicular (dash curves) polarization, respectively.

To understand the near-field response of nanoparticle aggregates to the CPL, analytic electromagnetic solutions to multi-sphere system simulations based on the generalized Mie theory (GMT) is performed^38^. The incident and scattered fields are expressed as a sum of vector spherical harmonics (VSH). The scattered fields from particles are calculated by the method of order-of-scattering^39^. The local field at any point is the sum of the incident field and coupling fields scattered from all of the particles (see Methods). We first consider a silver nanoparticle dimer (*R_1_* = *R_2_* = 40 nm, with the gap distance *d* = 1 nm, Fig. 1a) excited at *λ* = 532 nm with linear polarization. The highest near-field enhancement is obtained when the excitation is polarized parallel to the dimer axis (*y*-axis) which induces the coupled dipole in the dimer. For the perpendicular polarized excitation, there is nearly no field enhancement for this uncoupled dimer. Generally, the relation between the near field in the dimer gap and incident field can be expressed as:
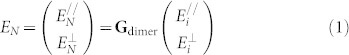
where *E_N_*^∥^ and *E_N_*^⊥^ are the near field components parallel and perpendicular to the dimer axis, respectively, and *E_i_*^∥^and *E_i_*^⊥^ are the corresponding components of the incident field. The enhancement tensor G_dimer_ can be written as:
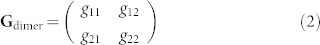


Then, we have to analyze each element in this tensor. As shown in Fig. 1b, the field in the gap is highly dependent on the incident polarization, hence, we have *g_11_* ≠ 0 and *g_12_* = 0 in this dimer case. Furthermore, at the surface of the metal, the enhanced near field in the junction is almost perpendicular to the surface irrespective of the incident polarization (white arrows in Fig. 1b). Then, both *g_21_* and *g_22_* are set to be zero. Hence, only the factor *g_11 = _|g_11_|e^iΔ^*^1^ is non-zero, where *Δ_1_* is the phase difference between the near field *E_N_*^∥^ and incident field *E_i_*^∥^, induced by the light scattering. The near-field enhancement *M*_dimer_ = |*E_N_*|^2^/|*E_i_*|^2^ = |*g_11_*|^2^.

When the incident light is circularly polarized, the near field in the dimer gap can be obtained by introducing the expression of the incident CPL into the formula (1),
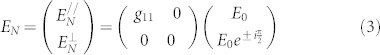
where “−” and “+” correspond to LCP and RCP incidences, respectively. Then we have the magnitude of the near field *|E_N_| = |g_11_| E_0_*, which is irrelevant to the optical rotation.

The case will be quite different if we introduce another particle into the dimer system. As shown in Fig. 1c, the right-angle trimer is formed by positioning a particle (*R* = 40 nm) on the right side of the 2^nd^ one with the distance *d* = 1 nm. Here we still consider the local field in the gap between the 1^st^ and 2^nd^ particles. As shown in Fig. 1d, the near-field enhancement for the perpendicular (*x*-axis) polarized excitation is comparable with the parallel (*y*-axis) polarized case at *λ* = 532 nm. The near-field enhancements for the two orthogonal polarizations as a function of polarization and wavelength are shown in Fig. 1e and 1f, respectively. Obviously, due to the strong coupling of the 3^rd^ particle with the dimer, the local field in the gap is insensitive to the incident polarization, especially in the wavelength range of 500 ~ 600 nm.

**Figure 2:**
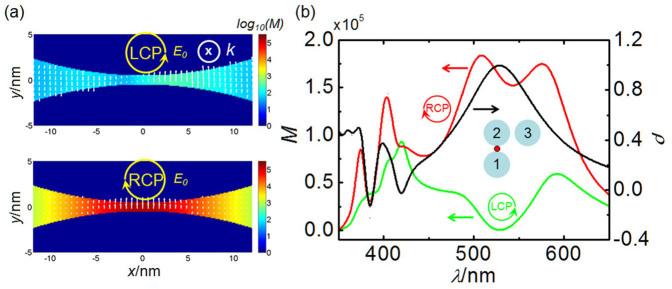
Local CD response of the right-angle trimer. (a) Electric field intensity distributions around the interparticle junction in the right-angle trimer excited by LCP and RCP at 532 nm, respectively. (b) Local field intensity and local CD response *ρ* (black curve, to the right axis) at the hotspot in the interparticle junction as a function of the incident wavelength. The green and red curves are for LCP and RCP excitations, respectively. The CD response reaches its maximum value of 0.998 at 532 nm.

For the CPL excitation, the relation between the scattered and incident field can still be depicted by formula (1) with a response tensor G_trimer_ for the trimer. Just as we did in the dimer case, it is still necessary to know the elements in G_trimer_. First, as the electromagnetic boundary relation still holds for trimer case, the polarization of near field in the gap is perpendicular to the metal surface (white arrows in Fig. 1d). Hence, we still have *g_21_* = *g_22_* = 0 for tensor G_trimer_. Second, different from the dimer case, the perpendicular *E_i_*^⊥^ could also result in significant enhancement *E_N_*^∥^, therefore both *g_11_* and *g_12_* are generally nonzero. So the near field for CPL excitations can be written as: 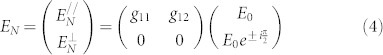
where *g_11 = _|g_11_|e^iΔ^*^1^, *g_12 = _|g_12_|e^iΔ^*^2^, and *Δ_2_* is the phase difference between the near field *E_N_*^⊥^ and incident field *E_i_*^⊥^. After simple derivations, the near-field enhancement for the CPL incidence can be obtained:
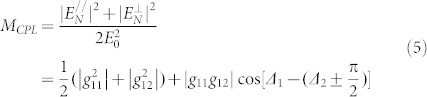


Obviously, *M_CPL_* depends highly on the field enhancement factors *|g_11_|* and *|g_12_|*, and especially the phase difference between *Δ_1_* and *Δ_2_*. For example, at *λ* = 532 nm, *|g_11_|*^*2*^ ≈ *|g_12_|*^*2*^ = 0.8*10^5^, and *Δ_1_* − *Δ_2_* ≈ π/2. The field enhancement at the gap for LCP and RCP excitation will be *M_LCP_* = 2.6*10^2^ and *M_RCP_* = 1.6*10^5^, respectively. The enhancement in the gap excited by the RCP light is much larger than the LCP case as shown in Fig. 2a, that is, the local CD. Actually, the local CD is the consequence of the near-field destructive/constructive interferences determined by the *Δ_1_* − *Δ_2_* when the factor g_11_*g_12_ are nonzero. Similar near-field interference mechanism had been used for controlling the surface plasmon polaritons in plasmonic waveguides^40^,^41^.

According to the definition of CD signals for far-field transmission or reflection^22^, we introduce a ratio *ρ* to evaluate the difference of near-field enhancement for incident LCP and RCP, which is defined as 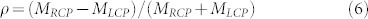


A giant local CD response *ρ* = 0.998 is obtained for the right-angle trimer at 532 nm. For other wavelengths, the partial interference results in the local CD spectrum shown by the black curve in Fig. 2b.

If a chiral molecule is situated in a symmetric dimer, the interaction between the molecule and nanoparticles would result in a far-field plasmonic CD^29^,^30^,^31^, although the hotspot in dimer gap does not have near-field CD response as discussed above. For the asymmetric timer case, the near-field intensity in the gap could have 1000 times difference depending on the handedness of light. This will add to the complexity of molecule-induced plasmonic chirality. It can be expected that the far-field CD signal, especially the term originated from the exciton-plasmon interaction, could be enhanced or weakened, determined by the combination of the corresponding positive or negative near-field CD and the optical rotatory dispersion of the molecule at surface plasmon resonance wavelength.

## Discussion

**Figure 3:**
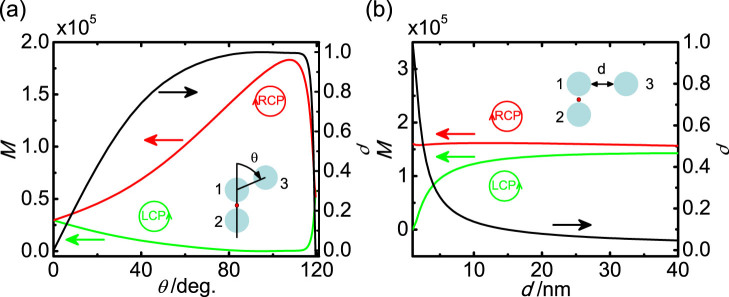
Local CD response as a function of the geometry. (a) Local field intensity and CD response at the hotspot as a function of the rotation of the 3^rd^ particle around the 2^nd^ particle. (b) Local field intensity and CD response at the hotspot as a function of the distance *d* between the 3^rd^ and 2^nd^ particle. The green and red curves are for LCP and RCP excitations, respectively. The CD response *ρ* is shown by the black curve to the right axis. The excitation wavelength is 532 nm.

The local CD is highly dependent on the geometry of the nanoantenna. Fig. 3a shows how the near-field enhancement *M* and CD response *ρ* change as the rotation of the 3^rd^ particle around the dimer. When *θ* = 0°, the linear trimer corresponds to the point group *D_∞h_*^42^,^43^. The perpendicular (*x*-axis) polarized incidence will excite weak coupled *π_u_* mode. Hence, similar to the dimer, the enhancement factor *g_12_* ≈ 0, and *M_LCP_* = *M_RCP_*, *i.e.*, *ρ* = 0. When the 3^rd^ particle is rotated around the dimer (*θ* = 0° ~ 90°), a *C_2v_* trimer is formed. In this configuration, both *A_1_* and *B_2_* plasmon modes^42^ can be excited and result in considerable field enhancement in the dimer gap irrespective to the incident polarization, *i.e.*, both *g_11_* and *g_12_* are nonzero. Consequently, the local CD response *ρ* grows with the increase of *θ* and reaches its maximum around 90°. After passing 90°, the 3^rd^ particle gets closer to the 1^st^ one and finally forms an equilateral trimer (*D_3h_* group) at *θ* = 120°. Both degenerate *E*′ modes in the *D_3h_* trimer can be excited by two orthogonal polarizations^43^. When the incident polarization is parallel to the 1^st^–2^nd^ dimer axis, three particles coupled each other strongly and result in the near-field enhancement in the particle gaps, i.e. a large factor *g_11_*. While for the perpendicular polarized incidence, the *E*′ mode is contributed by the dipolar coupling of 3^rd^ with 1^st^ and 2^nd^ particles, while the interaction between 1^st^ and 2^nd^ particles is rather weak. Hence, there is almost no near-field enhancement in the 1^st^–2^nd^ dimer gap, i.e., *|g_12_|* ≪ *|g_11_|*. Then, local CD response *ρ* drops rapidly to zero again when *θ* = 120°.

Apart from the structural asymmetry, *ρ* is also related to the magnitude of the coupling between 2^nd^ and 3^rd^ particles. As shown in Fig. 3b, when *d* is only several nanometers, both *g_11_* and *g_12_* are nonzero. According to the formula (5), *ρ* (black curve) is close to one, indicating a strong local CD response. The near-field enhancement for RCP excitation is much larger than the LCP case (green and red curves for LCP and RCP, respectively). As *d* increases, the 2^nd^–3^rd^ interparticle coupling becomes weaker, which causes *g_12_* dropping rapidly to zero. Correspondingly, *ρ* turns to be zero just as the dimer case.

**Figure 4:**
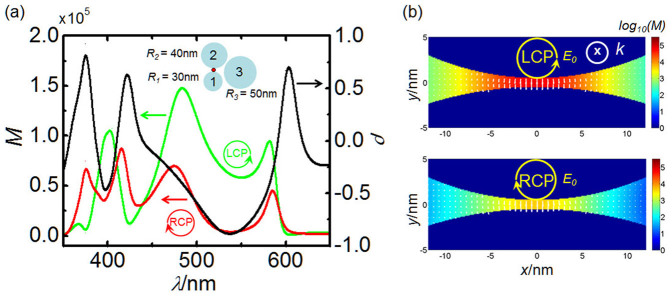
Local CD response of a non-identical trimer. (a) Local field intensity and CD response at the hotspot as a function of the incident wavelength. The green and red curves are for the LCP and RCP excitations, respectively. The CD response *ρ* is shown by the black curve to the right axis. (b) Electric field intensity distributions around the interparticle junction for the incident LCP and RCP, respectively. The wavelength of the excitation is 535 nm.

Actually, the local CD response widely exists in asymmetric nanoparticle antennas. In Fig. 4a, we consider a non-identical trimer with the radii 30 nm, 40 nm, and 50 nm for the 1^st^ to 3^rd^ particles, respectively, and a fixed surface-to-surface distance *d* = 1 nm. The electric field enhancement at the hotspot for LCP and RCP excitations are shown by green and red curves, respectively. Due to the asymmetric coupling between particles, the wavelength dependences of the near-field enhancement *g_11_* and *g_12_* become quite complex. Hence, the difference in the spectrum of the near-field enhancement for LCP and RCP will result in the negative CD response *ρ* = −0.89 at 535 nm, and positive *ρ* = 0.74 at 605 nm (black curve). At *λ* = 535 nm, the near field for LCP excitation will be much larger than the RCP case as shown in the Fig. 4b.

**Figure 5:**
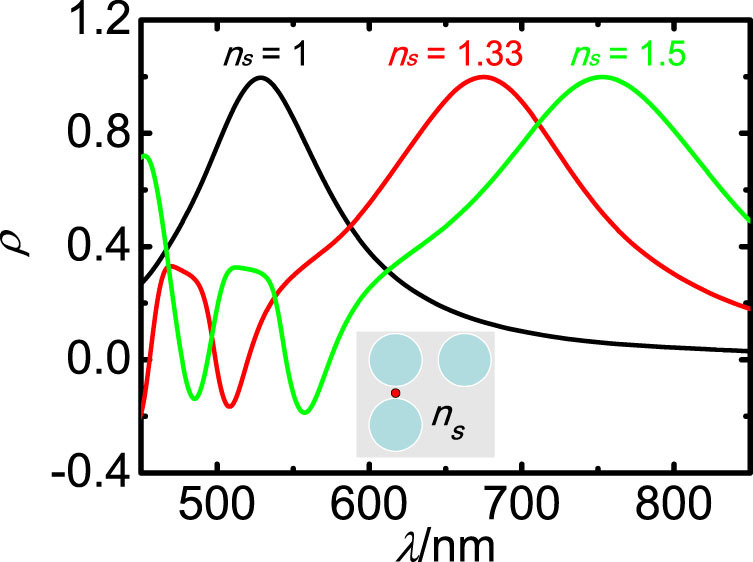
Local CD response of the right-angle trimer in different dielectric environment. Black, red and green curves are the trimer in air, water, and oil, with the refractive index *n_s_* = 1, 1.33, and 1.5, respectively.

As discussed above, the geometry and size determine the plasmonic modes excited in the nanoantenna, and consequently the local CD. Another important parameter influencing the surface plasmon coupling is the environment. Fig. 5 shows the spectrum of *ρ* for the right-angle trimer in air, water, and oil, with the refractive indexes *n_s_* = 1, 1.33, and 1.5, respectively. The peak of wavelength-dependent *ρ* has an obvious red-shift with the increasing of *n_s_*. Similar to the resonance shift in surface plasmon resonance sensors, the CD response in the gap in asymmetric structures is quite sensitive to the environment. The sensitivity factor, i.e., the shift in peak position per unit change in the refractive index of the surrounding medium, is 450 nm per refractive index unit, which is generally larger than usual sensitivity factor of localized surface plasmon peak^44^.

In conclusion, we have investigated the near-field response in silver nanoparticle aggregates under the excitation of circular polarized light. We found that, the near-field enhancement of the hotspot in a right-angle trimer is highly dependent on the polarization rotation, that is, local CD originated from the near-field interference in the strong coupled particle system. The local CD can be evaluated by the parameter *ρ*. It is obtained a nearly unity *ρ* in the right-angle trimer excited at *λ* = 532 nm, where the near-field excited by RCP is much larger than the LCP case. This phenomenon widely exists in non-identical trimers, and can be tuned by the rotation of the 3^rd^ particle and the interparticle distance. Furthermore, we found the CD response is quite sensitive to the environment and could be detected by the Raman signals of molecules, or fluorescence of quantum dots in one of the particle gaps. The manipulation of optical response through polarization and geometrical parameters opens possibilities for a wide range of applications, such as SERS^45^,^46^,^47^ or ROA substrates^22^,^48^, polarization sensitive devices^8^, sensors^36^ and bioapplications^18^, *etc*.

## Methods

Based on the generalized Mie theory^39^, the incident and scattered electromagnetic fields are expanded into vector spherical harmonics (VSH). The expansion coefficients for incident field are well known as Mie coefficients. The scattered electric fields for N-spheres system are:
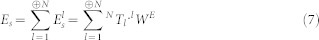
where *^l^W^E^* is the matrix of VSH, *^N^T_n_* is the scattering matrix for the N-spheres system:
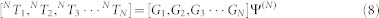


*G_N_* is the incident coefficient for *N^th^* sphere, ψ^(N)^ is called response matrix of the system (for details see Ref.^39^). The scattered electric field under circularly polarized excitation is then: 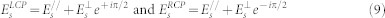
where *E_s_^LCP^*, *E_s_^RCP^*, *E_s_^//^* and *E_s_*^⊥^ are the scattered electric fields under LCP, RCP, parallel, and perpendicular polarized excitations, respectively. Dielectric data of silver come from the work of Johnson and Christy in Ref.^49^.

## Author Contributions

H.W. and Z.L. carried out the GMT simulations, and performed derivation of formulas. Z.L., H.Z., P.W. and S.W. interpreted and analyzed the data, and co-wrote the paper. Z.L., H.Z. and S.W. supervised the project. All authors discussed the results and commented on the manuscript.
